# Magneto‐Acoustic Field‐Induced Unstable Interface of Magnetic Microswarm

**DOI:** 10.1002/advs.202403039

**Published:** 2024-07-23

**Authors:** Rencheng Zhuang, Dekai Zhou, Junmin Liu, Xiaocong Chang, Guangyu Zhang, Longqiu Li

**Affiliations:** ^1^ State Key Laboratory of Robotics and System Harbin Institute of Technology Harbin Heilongjiang 150001 China; ^2^ Chongqing Research Institute Harbin Institute of Technology Chongqing 400722 China

**Keywords:** interfacial instability, magneto‐acoustic field, microswarm, two‐phase system

## Abstract

Research on the interfacial instability of two‐phase systems can help in gaining a better understanding of various hydrodynamic instabilities in nature. However, owing to the nonlinear and complex spatiotemporal dynamics of the unstable interface, the instability is challenging to control and suppress. This paper presents a novel interfacial instability of the magnetic microswarm induced by the competition between the destabilizing effect of magnetic field and the stabilizing effect of acoustic field. The physics underlying this novel phenomenon is discussed by analyzing the contributions of the external fields. Unlike previous studies, this study demonstrates that the instability is independent of the interfacial force or diffusion effect and can persist without dissipation over time. The manipulation of the unstable interface is further achieved by adjusting the configuration of the magneto‐acoustic system. This approach can be used in thermal encoding metamaterials and has great potential applications in systems where the instability is detrimental.

## Graphical abstract

The interfacial instability of two‐phase systems is a fascinating field of research due to their unique dynamic behaviors and interesting morphologies. A novel interfacial instability in a two‐phase system is reported. Suppression and manipulation of the unstable interface is achieved. By associating the findings with informatics, a reconfigurable thermal encoding metamaterial is designed and the information transmission is achieved.

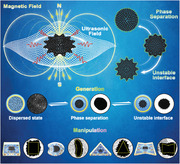

## 1 Introduction

The interfaces of two‐phase systems can exhibit spatial instability owing to the differences in the two‐phase properties (e.g., density or viscosity) or external stimuli (e.g., external field or mechanical vibration), resulting in complex dynamic behaviors and fascinating morphologies.^[^ ^1^, ^2^, ^3^, ^4^, ^5^ ^]^ The interfacial instability of two‐phase systems has been observed in various physical systems ranging from the celestial scale to the nanoscale. Examples include the supernova explosion in astrophysics,^[^ ^6^ ^]^ laser‐induced thermonuclear fusion in nuclear physics,^[^ ^7^ ^]^ and microbial bioconvection patterns in biology.^[^ ^8^ ^]^ Research on interfacial instability in two‐phase systems can help us better understand numerous hydrodynamic instabilities in nature.

Previous studies on interfacial instability have predominantly been conducted using miscible^[^ ^9^, ^10^, ^11^ ^]^ or immiscible^[^ ^12^, ^13^, ^14^ ^]^ fluid systems. It is commonly held that the interfacial instability is dominated by a competition between the destabilizing effects of external perturbations and the stabilizing effects of interfacial force or diffusion.^[^ ^15^, ^16^ ^]^ However, as the interfacial force and diffusion effect are inherent fluid properties, the interfacial instability is difficult to manipulate. Furthermore, unstable interfaces exhibit highly nonlinear and complex spatiotemporal dynamics, which poses greater challenges to control and suppress the instability.^[^ ^17^, ^18^ ^]^ In fact, instability is undesirable and detrimental to engineering applications such as film coating production,^[^ ^19^ ^]^ microfluidic device fabrication,^[^ ^20^ ^]^ and laser‐induced thermonuclear fusion.^[^ ^21^ ^]^ Therefore, it is critical to control and suppress the interfacial instability. To this end, previous studies have made considerable efforts by utilizing mechanical vibrations^[^ ^22^, ^23^ ^]^ and manipulating the geometry of fluid systems.^[^ ^24^, ^25^ ^]^ However, these methods are suitable only in the case of highly flexible in the design or setup of the device. To overcome these limitations, utilizing external (optical,^[^ ^26^ ^]^ electric,^[^ ^27^ ^]^ thermal,^[^ ^28^ ^]^ and magnetic^[^ ^29^ ^]^) fields to control interfacial instabilities has attracted extensive attention. Despite impressive progress, the results are largely restricted to theories and simulations. Flexible control and effective stabilization of the unstable interface remain key problems to be solved.

In this study, we identified a novel interfacial instability in a two‐phase system consisting of magnetic microparticles and the aqueous solution. This instability bears a surprising resemblance to the interfacial instabilities in two‐phase fluid systems, not only in interfacial morphologies but also in hydrodynamic features. However, contrary to common perception, this instability is independent of the interfacial tension or diffusion effect, but results from coupled external fields. Under the coupling action of magnetic and acoustic fields, phase separation occurs in the two‐phase system, resulting in the formation of a gear‐shaped microswarm with an oscillatory unstable interface. We discussed the physics underlying this novel phenomenon by analyzing the destabilizing effect induced by the magnetic field and the stabilizing effect induced by the acoustic field. Furthermore, the coupled external fields offer many unique advantages, such as on‐demand customization, flexible adjustment, and wireless control. Based on these advantages, we demonstrated that unstable interfaces can be suppressed and manipulated by changing the configuration of the external fields. In addition, by associating our finding with thermotic, we designed a reconfigurable thermal encoding metamaterial that can achieve the information transmission by manipulating microscopic thermal fields. The proposed method offers fascinating possibility for the design of smart functional devices and opens a new avenue to manipulate the unstable interface.

## 2 Results and Discussion

### 2.1 Principle of the Interfacial Instability

**Figure 1:**
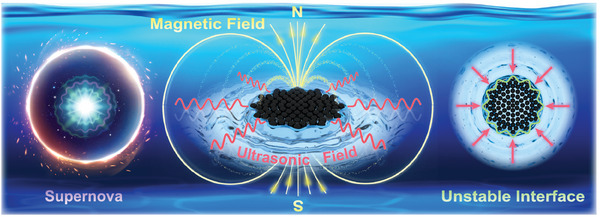
Schematic of interfacial instability of the two‐phase system under coupled external field analogous to the supernova explosion in astrophysics.

A two‐phase system consisting of magnetic microparticles and an aqueous solution underwent phase separation when subjected to an ultrasonic field (**Figure** **1**). Owing to the influence of acoustic radiation forces, the magnetic microparticles immediately aggregated into a swarm with a circular interface located at the pressure node of the standing wave. In this case, the circular interface of the swarm was time‐invariant, consistently maintaining its shape as the input acoustic field energy increased. Therefore, the circular interface of the swarm was stable. When an oscillating magnetic field perpendicular to the standing wave plane was superposed, the swarm evolved into a gear‐shaped formation. The gear‐shaped interface between the swarm and fluid exhibited periodic oscillatory behavior in response to the input magnetic field (Video S1, Supporting Information). The gear‐shaped interface of the swarm was unstable. As the magnetic field energy increased, the gear‐shaped interface continuously developed and eventually dissipated, analogous to the supernova explosion phenomenon in astrophysics.

In this study, the microswarm consisted of a large number of spherical γ‐Fe_2_O_3_ magnetic colloidal microparticles. During ultrasonic aggregation, phase separation occurred, creating a particle‐rich phase at the center of the experimental cell and a particle‐depleted phase at the edge. This led to an asymmetric distribution of microparticle concentrations within the cell. When an oscillating magnetic field was applied perpendicular to the plane of the two‐phase interface between the microswarm and the solution, the circular interface immediately evolved into an oscillatory unstable state. This interfacial instability can be associated with the Rayleigh–Taylor (RT) instability of two‐phase fluids, which occurs at the interface where a dense fluid displaces a lighter one under the action of gravity.^[^ ^30^ ^]^ However, different from the RT instability of the two‐phase fluid system, the gravity on the microswarm was balanced by the acoustic radiation force in our experiments. Furthermore, the surface tension between the microswarm and the surrounding solution as well as the diffusion effect of the microparticles were very weak and can be neglected. The microswarm with a gear‐shaped unstable interface was generated owing to the coupled action of the external fields. The magnetic force promoted the inversion of the density, whereas the acoustic force restricted the dissipation of the unstable interface.

**Figure 2:**
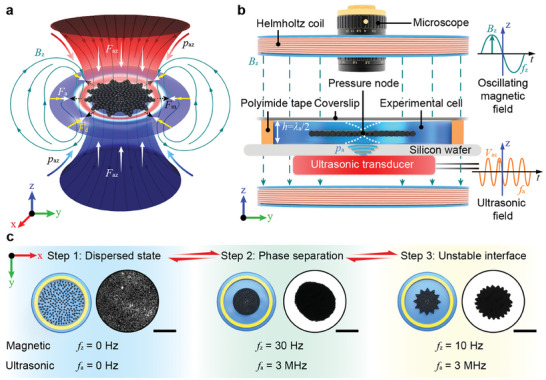
Force equilibrium condition, magneto‐acoustic system, and formation process of the oscillatory unstable interface. a) Schematic depiction of the force equilibrium conditions at the interface of the two‐phase system under the coupled action of magnetic and acoustic fields. b) Schematic of the magneto‐acoustic experimental system. c) Formation process of the gear‐shaped microswarm with an oscillatory unstable interface. The whole process consists of three steps, which are achieved by controlling the parameters of the magnetic and ultrasonic fields. Insets: Optical images of the two‐phase system consisting of magnetic microparticles. Scale bar: 500 µm.

We therefore analyzed the force equilibrium conditions at the interface of the two‐phase system under coupled magnetic and acoustic fields (**Figure** **2**a). Under the applied fields, the microswarm was confined to the center of the experimental cell owing to the action of the acoustic radiation force (*F*_az_) along the z‐axis. The magnetic force (*F*_m_) replaced gravity and drove the density inversion in the x‐y plane. This resulted in an instability at the interface between the microswarm and the solution. Owing to the in‐plane acoustic pressure gradient, the oscillatory unstable interface was limited and remained continuously in the linear stage as a consequence of the action of the transverse acoustic radiation force (*F*_a_). In addition, the unstable interface was subject to drag force (*F*_d_) during the oscillatory process.

### 2.2 System Design and the Formation Process of Interfacial Instability

**Figure 3:**
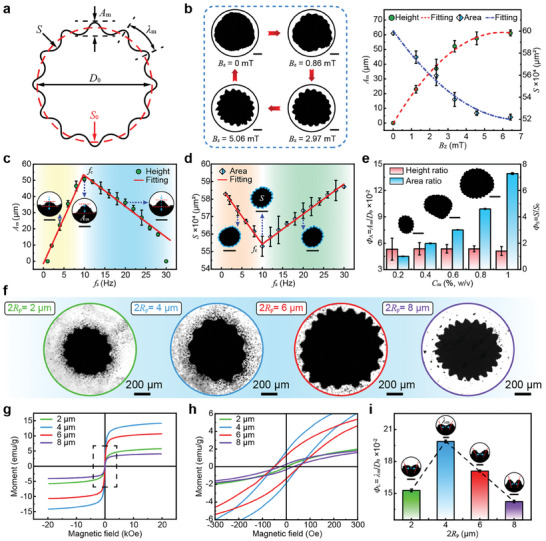
Dynamics of the unstable interfaces and corresponding swarm morphologies. a) Definition of several characteristic parameters of the unstable interface and swarm. b) Influence of the magnetic strength on maximum characteristic height *A*_m_ and minimum projected area *S* of the swarm (*f*_z_ = 10 Hz). Images showing microswarms under different magnetic strengths. Scale bar: 200 µm. c) Maximum characteristic height *A*_m_ versus magnetic frequency *f*_z_ (*B*_z_ = 3.71 mT). Insets: Images of a single tip of the unstable interface. Scale bar: 100 µm. d) Projected area *S* versus the magnetic frequency *f*_z_ (*B*_z_ = 3.71 mT). Insets: Images of microswarms. Scale bar: 500 µm. e) Influence of the microparticle concentration on normalized characteristic height ϕ_A_ and projected area ϕ_S_ (*f*_z_ = 10 Hz and *B*_z_ = 3.71 mT). Images showing microswarms at different concentrations. Scale bar: 500 µm. f) Images of swarms consisting of microparticles with different diameters and susceptibilities at the critical magnetic frequency (*B*_z_ = 3.71 mT). g) Measurement of magnetic properties of microparticles with different diameters. h) Enlarged view of the initial part of the hysteresis curve for microparticles with different diameters. i) Normalized characteristic wavelength ϕ_L_ versus diameter 2*R*_p_ of the microparticles at the critical magnetic frequency (*B*_z_ = 3.71 mT). Insets: Images of the characteristic wavelength of the unstable interface. Scale bar: 100 µm.

In this study, we conducted the experiment using a simple magneto‐acoustic system (Figure 2b and Experimental Section). In a typical process, the microparticles used in the experiment with a diameter of 8 µm (2*R*_p_ = 8 µm) and an effective magnetic susceptibility^[^ ^31^ ^]^ of χ_p_. The solution containing the microparticles with a mass concentration of *C*_m_ was added into the experimental cell. The formation of the gear‐shaped microswarm with an oscillatory unstable interface could be divided into three steps (Figure 2c; Video S2, Supporting Information). First, with no applied field, the microparticles were uniformly dispersed in the aqueous solution and exhibited an even distribution on the silicon substrate. Second, ultrasonic and oscillating magnetic fields were sequentially applied along the z‐axis. Under the ultrasonic field with an energy density^[^ ^32^ ^]^ of $E_{\mathrm{ac}}=p_{a}^{2}/4\rho_{m}c_{a}^{2}$ (where *p*_a_ is the acoustic pressure amplitude, ρ_m_ is the density of the solution, and *c*_a_ is the speed of sound in water), an ultrasonic standing wave (USW) was formed in the experimental cell. The height of the cell was equal to half of the ultrasonic wavelength (*h*  = λ_a_/2  = *c*_a_/2*f*_a_ ; where *h* is cell height, λ_a_ is ultrasonic wavelength, and *f*_a_ is ultrasonic frequency) and the diameter of the cell was *D*. The aqueous solution of microparticles exhibited phase separation, and the microparticles migrated toward the pressure node of the standing wave as a result of the acoustic radiation force (*F*_az_), which was derived from the acoustic pressure gradient (*p*_az_) along the z‐axis. Furthermore, the microparticles aggregated into a swarm with a circular interface on account of the transverse acoustic radiation force (*F*_a_). This phenomenon has been widely studied.^[^ ^33^, ^34^ ^]^ When a uniform magnetic field ***B***_z_(*t*)  = *B*_z_  · sin (2π*f*_z_ *t*) ***e***_z_ was continuously applied at a frequency of 30 Hz (where *B*_z_ is maximum magnetic field strength, *f*_z_ is oscillation frequency, *t* denotes time, and ***e***_z_ is the unit vector along the z‐axis), the microswarm was further agglomerated owing to the magnetic dipole‐dipole interactions.^[^ ^35^ ^]^ The ratio φ_*s*_ = *S*_0_/*S*_1_  [where *S*_0_ is the projected area of the swarm under the ultrasonic and static magnetic fields (**Figure** **3**a) and *S*_1_ is the projected area of the swarm under the ultrasonic field] of areas was ≈65.5% (Figure S1, Supporting Information). Finally, the magnetic field oscillation frequency was reduced from 30 to 10 Hz, and the circular microswarm evolved into a gear‐shaped pattern with an oscillatory unstable interface. Fast and reversible control of the two‐phase interface and swarm pattern can be achieved by varying the magnetic and ultrasonic field parameters.

### 2.3 Mechanisms and Dynamics of the Unstable Interfaceunstable Interface

When the magnetic microparticles in the swarm were subjected to an oscillating magnetic field, the chain‐shaped structures were formed.^[^ ^36^ ^]^ The individual chains assembled as building blocks to form the swarm. Due to the applied magnetic field was an oscillating field with time‐varying intensity, the microparticle chains were subjected to an alternating magnetic torque. The magnetic torque tended to align the microparticle chains parallel to the direction of the external magnetic field lines. Furthermore, under the action of the acoustic field, microparticle chains were subjected to an acoustic torque due to the axial acoustic radiation force acting on the microparticles. The acoustic torque tended to align the microparticle chains parallel to the nodal plane of the USW field. Therefore, the microparticle chains oscillated in a plane perpendicular to the nodal plane under the coupled action of the magnetic and acoustic torques. In this case, the dense microparticles periodically moved in and out of the lighter surrounding solution with a certain acceleration, resulting in the interfacial instability occurred at the interface of the two phases.

To characterize the interfacial instability, we analyzed the forces exerted on the microswarm in the nodal plane. The instantaneous height (*r*) of the unstable interface oscillated with time under the coupled action of magnetic, acoustic radiation, and drag forces (details are illustrated in Note S1, Supporting Information). The magnitude of the instantaneous height depended on the relative magnitude of the three forces. Therefore, we first experimentally investigated the influence of the magnetic field strength on the unstable interface and the morphology of the swarm while maintaining a constant magnetic frequency (*f*_z_) of 10 Hz. Due to the height of the unstable interface was time‐invariant, we focused on the maximum value of the instantaneous height (*A*_m_), which corresponds to the minimum projected area (*S*) of the swarm (Figure 3a). As the maximum magnetic field strength (*B*_z_) increased, the maximum characteristic height of the tips increased due to the increasing magnetic force, while the projected area of the swarm decreased because of the increasing magnetic dipole‐dipole forces^[^ ^37^, ^38^ ^]^ (Figure 3b; Video S3, Supporting Information). However, when the magnetic field strength was too high, the drag force increased significantly and limited the growth of the tips. Furthermore, the experimental results demonstrate that the transformation of the interface can be accomplished by tuning the magnetic field strength. The two‐phase interface switched between the stable circular state and unstable gear state by changing the amplitude of the sinusoidal signal fed into the coil. We have characterized the magnetic strength generated by the three‐axis Helmholtz coils when sinusoidal signals of different amplitudes were applied (Note S2 and Figure S2a–c, Supporting Information).

In addition, the condition of the microparticle chains has been reported to be strongly dependent on the magnetic frequency.^[^ ^39^ ^]^ Therefore, the magnetic field frequency was another important factor affecting the interfacial instability. We maintained the magnetic strength of 3.71 mT and investigated the influence of the magnetic frequency on the interfacial instability and morphology of the swarm (Figure 3c,d). The experimental results showed that a critical frequency (*f*_c_) existed for the unstable interface. This frequency was related to the maximum rotational angular velocity at which the chains broke apart, which can be determined by the counterbalance relationship between the torques exerted on the microparticle chains (details are illustrated in Note S3, Supporting Information). As the frequency in the low‐frequency band (yellow and orange regions in Figure 3c,d) increased, the angular velocity of the rotational microparticle chains increased. This promoted density inversion and interfacial instability, resulting in an increase in the maximum characteristic height and a decrease in the projected area. When *f*_z_ > *f*_c_ (blue and green regions in Figure 3c,d), the chains no longer followed the magnetic frequency on account of the marked increase in the drag torque. In this case, the gear‐shaped unstable interface gradually disappeared, and the swarm expanded into a circular formation (Video S4, Supporting Information). Thus, it is demonstrated that the manipulation of the interfacial instability and swarm morphology can be achieved by controlling the magnetic field frequency. We further investigated the influence of the microparticle concentration on the interfacial instability. We quantified this process using the normalized characteristic height ϕ_A_ = *A*_m_/*D*_0_  [where *D*_0_ is the initial diameter of the microswarm under the ultrasonic and static magnetic field along the z‐axis (Figure 3a)], and the normalized projected area ϕ_S_ = *S*/*S*_0_. The experimental results indicated that the normalized projected area of the microswarm was proportional to the solution concentration on account of the increase in the microparticle numbers. However, the increased concentration had no effect on the normalized characteristic height of the unstable interface owing to the proportional increase in the unstable interface height and the swarm diameter (Figure 3e; Video S5, Supporting Information).

In addition to controlling the external magnetic field, changing the geometric parameters and magnetic properties of the microparticles can also affect the contribution of the magnetic field to the interfacial instability. Therefore, we investigated the interfacial instability of the two‐phase system consisting of microparticles with different diameters and susceptibilities (Figure 3f; Video S6, Supporting Information). We characterized the magnetic properties of the different microparticles (Figure 3g,h) using a magnetometer (Experimental Section). The initial magnetization curve of microparticles with the different diameters is shown in Figure S3 (Supporting Information). The experimental data indicated that the effective susceptibility (χ_p_) of the microparticles underwent a process of first increasing and then decreasing as the diameter of the microparticles (2*R*_p_) increased. The microparticles with a diameter of 4 µm have the largest effective susceptibility. Because the geometric and magnetic properties of the microparticles could affect the critical frequency (*f*_c_) of the unstable interface, we examined the influence of the magnetic frequency (*f*_z_) on the unstable interface and the morphology of the swarm consisting of different microparticles. The experimental results showed that the critical frequency of the unstable interface increased with the increasing effective susceptibility of the microparticles. In addition, the microparticles with a diameter of 4 µm were observed to have the highest critical frequency (Figure S4a,b, Supporting Information). Furthermore, the swarm consisting of microparticles with a higher effective susceptibility was associated with a higher characteristic height and smaller projected area at the critical frequency (Figure S4a,b, Supporting Information). This finding is mainly owing to the increase in the susceptibility leading to the stronger magnetic force and magnetic dipole‐dipole interactions acting on the microparticles. Interestingly, changes in the susceptibility of the microparticles led to different characteristic wavelengths λ_m_ of the unstable interface. The characteristic wavelength was defined as the distance between two adjacent tips (Figure 3a). We introduced a normalized wavelength parameter ϕ_L_ = λ_m_/*D*_0_  to describe the characteristic wavelength of the unstable interface. Figure 3i shows a linear increase in the normalized characteristic wavelength of the unstable interface versus the effective susceptibility of the microparticles. In addition, the normalized characteristic wavelength was independent of the magnetic frequency (Figure S5, Supporting Information). This indicated that the unstable interface did not have an obvious dispersion relationship.

### 2.4 Manipulation of the Unstable Interface

**Figure 4:**
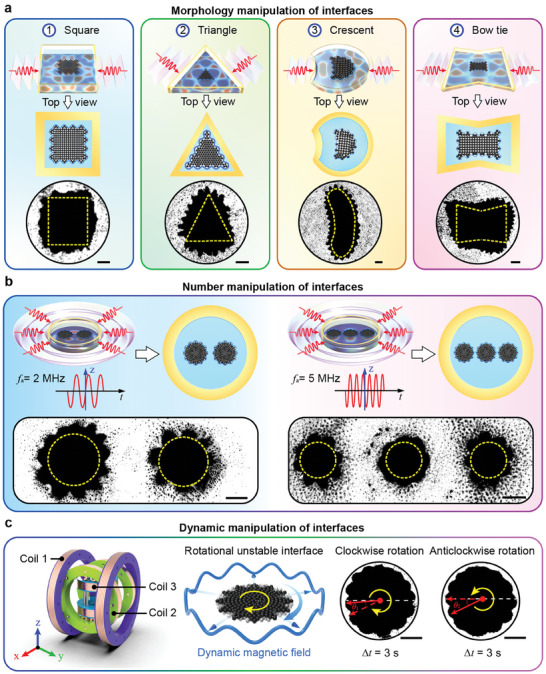
Static and dynamic manipulation of the unstable interfaces. a) Morphology manipulation of the unstable interface (squares, triangles, crescents, and bow ties) by changing the shape of the polyimide tape. Images showing the unstable interface and morphology of swarms under different acoustic fields and the oscillating magnetic field. Scale bar: 200 µm. b) Number manipulation of the unstable interface within a single experimental cell. The height of the polyimide tape used in the experiment was *h* = 375 and 150 µm, respectively. The ultrasonic frequency was *f*_a_ = 2 and 5 MHz, respectively. Images showing the unstable interface and morphology of swarms under different acoustic fields and the oscillating magnetic field. Scale bar: 200 µm. c) Dynamic manipulation of the unstable interface. Images showing the clockwise and anticlockwise rotational motion behavior of the gear‐shaped microswarm. The magnetic strengths *B*_z_ = 3.71 mT and *B*_x_ = *B*_y_ = 0.40 mT. The rotational angles θ_1_ = 16.2°and θ_2_ = 25.1°. Scale bar: 500 µm.

Acoustic‐based manipulation techniques have attracted considerable attention due to their advantages such as tunability, simplicity, and contactless.^[^ ^40^, ^41^, ^42^ ^]^ In this study, we achieved the static manipulation of the unstable interface by controlling the ultrasonic field. Specifically, this involved controlling the morphology and number of unstable interfaces within the experimental cell. We first analyzed the USW field in the experimental cell and simulated acoustic pressure, acoustic radiation force, and acoustic streaming fields in the nodal plane (Note S4 and Figure S6, Supporting Information). According to the simulation results, the microparticles moved toward the acoustic pressure antinodes in the nodal plane owing to the in‐plane acoustic radiation force and acoustic streaming, which pointed to the antinodes.^[^ ^43^, ^44^ ^]^ Furthermore, the simulation results indicated that the formation of the acoustic pressure field in the nodal plane significantly depended on the polyimide tape geometry. This affected the transverse acoustic wave propagation within the standing wave plane and the interface morphology. Based on this concept, we developed an acoustic platform to manipulate the morphologies of the unstable interfaces using square‐, triangle‐, crescent‐, or bow‐tie‐shaped polyimide tape (Experimental Section). We simulated the acoustic pressure field in the experimental cell nodal plane at an acoustic frequency *f*_a_ of 3 MHz for the different tape shapes (Figure S8, Supporting Information). To validate the simulation results, we conducted interfacial manipulation experiments using the aforementioned acoustic platform, in which an oscillating magnetic field was applied along the z‐axis. The experimental results showed that the interface morphology could be transformed into different formations, such as squares, triangles, crescents, or bow ties, depending on the shape of the polyimide tape (**Figure** **4**a). It should be noted that the oscillatory interfacial instability also occurred at the two‐phase interface when an oscillating magnetic field was superposed. This instability was independent of the interface morphology (Video S7, Supporting Information). We further achieved the number manipulation of unstable interfaces by adjusting the acoustic pressure field in the nodal plane. To control the number of acoustic pressure nodes within the nodal plane without affecting their morphology, we changed the height of the ring‐shaped polyimide tape, which corresponded to the different frequencies of the standing wave. We used polyimide tape with a height of 375 µm, which corresponded to a USW frequency of 2 MHz. The simulation results showed that there were two adjacent acoustic pressure nodes at the center of the nodal plane (Figure S9a, Supporting Information). To validate our numerical predictions, we performed experiments using an acoustic platform with experimental cells of different heights. Two microswarms appeared in the experimental cell, as predicted by the simulation (Figure 4b). In addition to the case demonstrated with two interfaces, the acoustic platform could be extended to include different numbers of interfaces. When the acoustic platform was constructed using polyimide tape with a height of 150 µm, it corresponded to a USW frequency of 5 MHz. According to the simulation results, three adjacent acoustic pressure nodes were formed at the center of the nodal plane (Figure S9b, Supporting Information). The experimental results showed that three microswarms formed in the experimental cell, which agreed well with our numerical prediction (Figure 4b). In addition, oscillatory interfacial instability occurred when the oscillating magnetic field was superposed, which was not affected by the number of interfaces (Video S8, Supporting Information).

Furthermore, by changing the configuration of the magnetic field, we achieved the dynamic manipulation of unstable interfaces. We simultaneously fed sinusoidal signals to the three‐axis Helmholtz coils and created a dynamic magnetic field, consisting of a rotating magnetic field ***B***_xy_ = ***B***_x_  +  ***B***_y_ = *B*_x_  · sin (2π*f*_xy_ *t*) ***e***_x_ + *B*_y_ · sin (2π*f*_xy_ *t*) ***e***_y_ (where *B*_x_ and *B*_y_ are the maximum magnetic field strengths, and ***e***_x_ and ***e***_y_ are the unit vectors along the x‐ and y‐axis, respectively) and an oscillating magnetic field ***B***_z_ along the z‐axis. The visualization of the superposition of the magnetic field over a cycle is shown in Figure S10 (Supporting Information). The magnetic frequencies of the in‐plane rotating field *f*_xy_ and out‐of‐plane oscillating field *f*_z_ are 1 and 10 Hz, respectively. The microswarm exhibits clockwise (0.09 rad s^−1^) and anticlockwise (0.15 rad s^−1^) rotational motion behaviors under the magnetic field. It is necessary to note that the oscillatory unstable interface existed stably during the rotational process owing to the action of the out‐of‐plane oscillating magnetic field (Figure 4c; Video S9, Supporting Information). Furthermore, the rotational direction of the unstable interface can be quickly transferred by changing the rotational direction of the in‐plane rotating magnetic field, which demonstrated that the unstable interface exhibited good dynamic controllability.

### 2.5 Thermal Field Manipulation and Information Transmission

**Figure 5:**
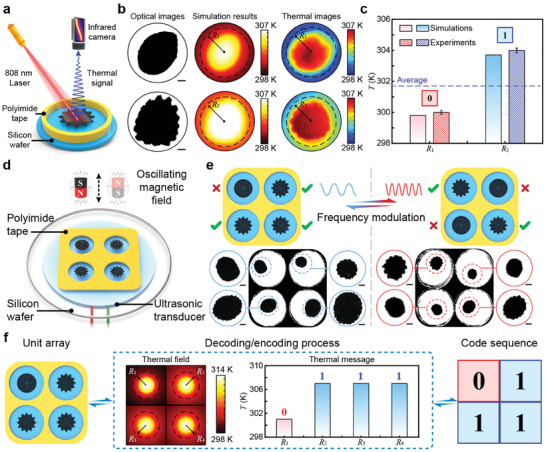
Thermal field manipulation and reconfigurable thermal encoding metamaterial. a) Schematic of the thermal measurement platform. The 808 nm laser was used as the light heat and an infrared camera was used to measure the thermal field distribution of the swarm under light irradiation. b) The optical images, thermal simulation results, and thermal images of the circular‐ and gear‐shaped microswarms. Scale bar: 100 µm. c) The thermal coding rules of the reconfigurable microswarms. The average temperature on equivalent‐radius circles of the circular‐ and gear‐shaped microswarms was used as the reference value. The average temperature on the equivalent‐radius circle of the microswarm was higher (lower) than the reference value can be recorded as 1 (0). d) Schematic of the magneto‐acoustic composite unit system. e) Images showing the reconfigurable manipulation of the interfaces and morphologies of microswarms in the unit array. The enlarged views of the microswarms in each experimental cell are in the blue and red circles. Scale bar: 200 µm. f) The decoding and encoding process of the reconfigurable thermal encoding metamaterial.

On‐demand thermal field manipulation has attracted tremendous attention and has been vigorously developed in the fields of energy, environment, communication, electronics, and biomedicine.^[^ ^45^, ^46^ ^]^ Due to the advantages of both the fluidity of liquids and the high thermal conductivity of solids, solid‐liquid two‐phase systems have great potential for dynamic manipulation of the thermal field over a wide range of temperatures.^[^ ^47^, ^48^ ^]^ Here, we investigated the feasibility of the proposed microswarms and their interfacial control methods for dynamic manipulation of microscopic thermal fields and demonstrated their potential application in information transmission. Due to the thermal conductivity of the liquid phase is far less than that of the solid phase, the circular‐ and gear‐shaped microswarms will exhibit different thermal responses under the same heat sources. Therefore, by controlling the interfaces of the microswarms, we can manipulate the local microscopic thermal field around the swarms. Furthermore, the different thermal responses and thermal field distribution can be associated with informatics. To demonstrate our purpose, we designed a thermal measurement platform. The thermal field distribution of the microswarms was measured using an infrared camera (**Figure** **5**a). An 808 nm laser was used as the heat source. To increase the heat energy generated by microswarms under the irradiation of light, the microparticles were modified using Au nanoparticles.

We first simulated and compared the thermal field distribution of microswarms with different interfaces under the irradiation of the near‐infrared light at *t* = 1 s (Figure 5b; Note S5, Supporting Information). To transform the thermal messages hidden in the thermal field distribution to the codes, we defined an equivalent radius *R* = 0.55*D*_0_ and calculated the average value of the temperature on the equivalent‐radius circle. The average temperatures on equivalent‐radius circles of gear‐shaped microswarms *T*_1_ are generally higher than those of circular‐shaped microswarms *T*_2_. Therefore, we can set a reference value, which is the average of *T*_1_ and *T*_2_. The microswarm with higher average temperature on the equivalent‐radius circle than the reference value will be recorded as 1, while the microswarm with lower average temperature will be recorded as 0 (Figure 5c). Furthermore, we measured the thermal field distribution of the microswarms and extracted the thermal messages (Figure 5b,c). The experimental results indicate that the thermal field distribution of the microswarms with circular‐ or gear‐shaped interfaces have significant differences. The average temperature on the equivalent‐radius circle of the gear‐shaped microswarm are higher than that of the circular‐shaped microswarm at *t* = 1 s, which agreed well with the simulation results. Therefore, the thermal message can be well described as the binary code.

Based on the coding rules, we proposed a reconfigurable thermal encoding metamaterial to convey the information with the BCD code. We designed a magneto‐acoustic composite unit system, containing of the polyimide tape with four experimental cells (Figure 5d and Experimental Section). By modulating the acoustic frequency, we can control the relative strength of the acoustic and magnetic forces, thus manipulating the interface and morphology of the microswarms in each experimental cell of the unit array. Based on the proposed magneto‐acoustic composite unit system, we achieved the reconfigurable manipulation of unstable interfaces in four experimental cells (Figure 5e). By using the near‐infrared light as the heat source and using the infrared camera to collect the thermal messages, the proposed magneto‐acoustic composite unit system can serve as the reconfigurable thermal encoding metamaterial to convey messages with the BCD code. Here, we used a code sequence (0111) and analysis the thermal field of the unit array to illustrate the decoding and encoding process (Figure 5f). By extracting thermal messages from the thermal field of the unit array at *t* = 1 s, the code sequence can be decoded from the unit array. In contrast, when we reversed this process, the code sequence was recorded in the unit array in the form of thermal messages. Therefore, the information can be well transferred using the proposed magneto‐acoustic composite unit system. Our approach provides excellent platforms for microscopic thermal field manipulation and may inspire new ideas for the design of smart functional devices.

## 3 Conclusion

In this paper, we report a novel interfacial instability in a two‐phase system. In contrast to the previously reported interfacial instability, the instability identified in this study was caused by the coupling action of magnetic and acoustic fields and was independent of the interfacial tension or diffusion effect. We analyzed the competition between the destabilizing effect of the magnetic force and the stabilizing effects of the acoustic radiation and drag forces. We defined several characteristic parameters to describe the unstable interfaces and investigated in detail the influence of the magnetic field on the unstable interfaces. Furthermore, we examined the effect of the microparticle properties on the unstable interfaces. Owing to the advantages of flexible and wireless control, the static and dynamic manipulation of unstable interfaces could be realized by adjusting the external fields. The findings reveal the interactive relationship between the coupled external fields and the unstable interface and provide a novel concept for stabilization and control of interfacial instability.

The experimental results revealed a new type of unstable interface, which was induced by the coupled interaction between the magnetic, acoustic radiation, and drag forces exerted on the microparticles. The magnetic force promoted the generation of the unstable interface, whereas the acoustic and drag forces restricted its dissipation. The interfacial tension and diffusion effect were notably weak in the experiment, which can be negligible (Note S1, Supporting Information). This phenomenon is similar to the Rosensweig instability of a ferrofluid droplet confined in a Hele–Shaw cell under a radial magnetic field. However, in this work, we constructed the confined domain with the external ultrasonic field instead of the Hele–Shaw cell, which allowed us to flexibly control the unstable interfaces and morphologies of microswarms by modulating the field parameters. Furthermore, unlike the magnetic‐field configurations reported in the study,^[^ ^49^ ^]^ we applied an oscillating magnetic field perpendicular to the microswarm. The magnetic field strength along the radial direction was much smaller than that along the z‐axis, and the region of the uniform magnetic field in the x‐y plane was much larger than the projected area of the microswarm (Note S2 and Figure S2d–f, Supporting Information). Therefore, no radial magnetic‐field gradient existed. Furthermore, the interfacial instability observed in our experiment can be associated with the secondary wave of the labyrinthine instability.^[^ ^50^ ^]^ However, labyrinthine instability was not observed in our experiment. This can be attributed to a weak diffusion effect. In addition, owing to the large diameter of the microparticles, the local microconvection^[^ ^51^ ^]^ at unstable interfaces was very weak. Only when the swarm consisted of microparticles with a diameter of 2 µm could we observe local microconvection, which was ejected from the tip of the unstable interface and injected into the depression. Our findings are different from those of existing studies on unstable interfaces and can help us better understand the fundamentals of interfacial instability.

In conclusion, we theoretically and experimentally studied the unstable interfaces induced by coupled external fields. We demonstrated the physics underlying the phenomenon by analyzing the interaction between the coupled external fields and the two‐phase interface. The proposed unstable interfaces could be controlled and suppressed by adjusting the characteristics of the external energy sources. Furthermore, we demonstrated the application of the proposed method in microscopic thermal field manipulation and information transmission. Further investigation should focus on conducting more quantitative experiments to develop physical models that characterize the evolution of unstable interfaces induced by coupled fields. In addition, there is enormous scope for progress in designing more combinations and configurations of external fields to achieve the flexible and robust control of the unstable interfaces. We envisage that control of the interfacial instability via the external physical fields can find a wide range of applications in systems where the instabilities are detrimental and the design of smart functional devices. From a broader perspective, the exploration of the field‐induced interfacial instability represents an important advance in our understanding of instabilities at two‐phase interfaces in nature.

## 4 Experimental Section

### Materials

Spherical γ‐Fe_2_O_3_ microparticles with different diameters (2, 4, 6, and 8 µm) were purchased from Aladdin Chemistry (Shanghai, China). Prior to the experiments, the purchased microparticles were washed three times with deionized water. The microparticles were then dispersed in deionized water and subjected to an ultrasonic bath for 3–5 min for uniform dispersion. The microparticles were modified with the Au nanoparticles in the thermal field measurement experiments. The Au nanoparticles were synthesized according to the previously reported work.^[^ ^52^ ^]^

### Experimental Devices and Data Analysis

The unstable interfaces and microswarms were observed under an optical microscope (Olympus BX53 with a 4× objective). Optical images were recorded by a video capture device (Teledyne FLIR Grasshopper3 USB3). Feature extraction was performed on some of the optical images, and only the relevant parts (the microswarms and their interfaces) were retained. The oscillations of the unstable interfaces were captured using a high‐speed camera (Phantom VEO E310L). An 808 nm laser with a power of 100 W cm^−2^ was used as the heat source and an infrared camera (FLIR X6520sc) with the macro lens was used to measure the thermal field distribution of the microswarms. Experimental data analysis was performed using ImageJ and Origin software. All data were calculated by averaging at least ten different measurements of the unstable interfaces at five time points, and the error bars represented the standard deviation of the averaged values at five time points.

### Preparation of the Magneto‐Acoustic System

The magneto‐acoustic system consisted of a magnetic‐field‐generating part and an ultrasonic‐field‐generating part. The oscillating magnetic field was generated using a set of three‐axis Helmholtz coils. The coils were connected to a function generator (Keysight Technologies 33500B) and a power amplifier (Aigtek Technologies ATA‐309). The sinusoidal signal was monitored by an oscilloscope (Keysight Technologies DSOX4024A). An ultrasonic transducer (STEMiNC SM111) with a 12‐mm diameter and 3‐MHz resonant frequency was used to generate the ultrasonic field. The ultrasonic transducer was attached to a silicon wafer that served as the substrate and transmitted the acoustic waves. A piece of ring‐shaped polyimide tape with a 250‐µm thickness was pasted on the silicon wafer to construct the experimental cell. The inner and outer diameters of the polyimide tape were 3 and 12 mm, respectively. A coverslip was overlaid on the polyimide tape to reflect the acoustic waves and generate a USW field. A function generator was connected to the ultrasonic transducer to generate a sinusoidal signal. In a typical process, the applied magnetic field had a frequency of 10 Hz and strength of 3.71 mT. The sinusoidal signal fed into the ultrasonic transducer had a frequency of 3 MHz and a peak‐to‐peak voltage of 10 V.

### Measurement of the Magnetic Properties

The magnetic properties of the microparticles were measured using a vibrating‐sample magnetometer (Lake Shore 7404). The magnetometer can detect changes in the magnetic flux caused by the mechanical sliding of the sample through a superconducting coil. By analyzing the measurement results, the hysteresis curve was obtained and calculated the magnetic susceptibility of the microparticles (Note S2, Supporting Information).

### Preparation of the Acoustic Experimental Platform

For the acoustic platform, acoustic and magnetic fields were applied in the same manner as for the magneto‐acoustic system. In contrast to the magneto‐acoustic system, the acoustic platform consisted of different shapes of the polyimide tapes with a 250‐µm thickness. The detailed geometric parameters of the different polyimide tapes are shown in Figure S7 (Supporting Information). Ultrasonic transducers (STEMiNC SM111) with resonant frequencies of 2 and 5 MHz were used to investigate the influence of the height of the experimental cell on the unstable interfaces. Two pieces of ring‐shaped polyimide tape with thicknesses of 375 and 150 µm were used. The inner and outer diameters of the polyimide tape were 3 and 12 mm, respectively. The sinusoidal signal fed into the ultrasonic transducer had a peak‐to‐peak voltage of 10 V and frequencies of 2 and 5 MHz.

### Preparation of the Magneto‐Acoustic Composite Unit System

For the magneto‐acoustic composite unit system, acoustic and magnetic fields were applied in the same manner as for the magneto‐acoustic system. In contrast to the magneto‐acoustic system, the polyimide tape with a 250‐µm thickness used in the composite unit system contained four experimental cells. The side length of the polyimide tape was 8 mm and the diameter of the experimental cell was 2 mm. An ultrasonic transducer (STEMiNC SM111) with a 12‐mm diameter and 3‐MHz resonant frequency was used to generate the ultrasonic field, which can cover the four experimental cells.

### Numerical Simulations

The numerical simulation was performed using commercial finite element analysis software COMSOL Multiphysics 5.5. The magnetic field was simulated using the magnetic fields module. The acoustic pressure, acoustic radiation force, and acoustic streaming fields were computed using the fully coupled pressure acoustics and creeping flow modules. The thermal field distributions of the microswarms under the irradiation of light were computed based on the heat transfer equations. More details of the numerical models and the related equations can be found in Notes S1–S5 (Supporting Information).

## Conflict of Interest

The authors declare no conflict of interest.

## Supporting information

Supporting Information

Supplemental Video 1

Supplemental Video 2

Supplemental Video 3

Supplemental Video 4

Supplemental Video 5

Supplemental Video 6

Supplemental Video 7

Supplemental Video 8

Supplemental Video 9

## Data Availability

The data that support the findings of this study are available from the corresponding author upon reasonable request.
