# Supplementary material for: Magneto‐Acoustic Field‐Induced Unstable Interface of Magnetic Microswarm
Source: Adv Sci (Weinh). 2024 Jul 23;11(36):2403039. doi: 10.1002/advs.202403039 (PMC11423188; doi:10.1002/advs.202403039)
Supplement: Supplementary file 1 — Supporting Information [file ADVS-11-2403039-s003.pdf]

ADVANCED SCIENCE
Open Access

Supporting Information

for *Adv. Sci.*, DOI 10.1002/advs.202403039

Magneto-Acoustic Field-Induced Unstable Interface of Magnetic Microswarm

Rencheng Zhuang, Dekai Zhou, Junmin Liu, Xiaocong Chang, Guangyu Zhang and Longqiu Li**

Supporting Information

for *Adv. Sci.*, DOI:

Magneto-acoustic field-induced unstable interface of magnetic microswarm

Rencheng Zhuang,[‡] Dekai Zhou,[‡,] * Junmin Liu, Xiaocong Chang,

Guangyu Zhang, and Longqiu Li*

Supporting Information

Magneto-acoustic field-induced unstable interface of magnetic microswarm

Rencheng Zhuang,[‡] Dekai Zhou,[‡,] * Junmin Liu, Xiaocong Chang, Guangyu Zhang, and Longqiu Li*

R. Zhuang, D. Zhou, J. Liu, X. Chang, G. Zhang, L. Li

State Key Laboratory of Robotics and System

Harbin Institute of Technology

Harbin, Heilongjiang 150001, China

E-mail: dekaizhou@hit.edu.cn; longqiuli@hit.edu.cn

D. Zhou, X. Chang

Chongqing Research Institute

Harbin Institute of Technology

Chongqing, 400722, China

‡ These authors contributed equally to this work.

* Corresponding author.

The PDF file includes:

Note S1. Details of the forces exerted on the microswarm in the nodal plane.

Note S2. Magnetic property calculations of the microparticles and the numerical simulation model of the magnetic field.

Note S3. Details of the torques exerted on the microparticle chains.

Note S4. The numerical simulation model of the acoustic field.

Note S5. Details of the thermal field distribution simulation of the microswarms.

Figure S1. The changes in the projected area of the microswarm during the formation process of the circular interface.

Figure S2. Schematic illustration of three-axis Helmholtz coils and characterization of the magnetic field.

Figure S3. The initial magnetization curve of microparticles with different diameters.

Figure S4. The influence of the magnetic frequency on the unstable interface and the morphology of the swarm consisting of different microparticles.

Figure S5. The normalized characteristic wavelength of unstable interfaces versus the magnetic frequency, where microparticle diameters are 2, 4, 6 and 8 μm.

Figure S6. Illustration of the acoustic simulation model and the simulated acoustic pressure, acoustic radiation force and acoustic streaming fields at an acoustic frequency of 3 MHz.

Figure S7. The geometric parameters of different polyimide tapes used in the experiment.

Figure S8. Numerical simulation results of the acoustic pressure field in the experimental cell nodal plane for different shapes of the polyimide tapes.

Figure S9. Numerical simulation results of the number manipulation of the unstable interfaces.

Figure S10. Visualization of the dynamic magnetic field. The magnetic strength was normalized.

Figure S11. The zero-order (J_0) and first-order (J_1) Bessel function of the first kind.

Figure S12. The linear fit of the magnetic property measurement data for microparticles with different diameters reveals the magnetic susceptibility from the slope.

Figure S13. The geometric parameters of the Helmholtz coil used in the magnetic simulation.

Table S1. Material and field parameters used for the numerical simulations.

Table S2. Geometric parameters used for the numerical simulations.

Supplementary References

Other Supplementary Material for this manuscript includes the following:

Video S1 (.mp4 format)**.** The oscillatory unstable interface of the gear-shaped microswarm. The video was captured using a high-speed camera with the sampling rate of 7000 fps.

Video S2 (.mp4 format)**.** The formation process of the gear-shaped microswarm with oscillatory unstable interface. The applied magnetic strength was $B_z = 3.71\,\mathrm{mT}$ and acoustic frequency was $f_a = 3\,\mathrm{MHz}$.

Video S3 (.mp4 format)**.** The gear-shaped microswarm and its unstable interface under different magnetic strengths. The applied magnetic frequency was $f_z = 10\,\mathrm{Hz}$.

Video S4 (.mp4 format)**.** The gear-shaped microswarm and its unstable interface under different magnetic frequencies. The applied magnetic strength was $B_z = 3.71\,\mathrm{mT}$.

Video S5 (.mp4 format)**.** The gear-shaped microswarms and their unstable interfaces under different concentrations. The applied magnetic strength was $B_z = 3.71\,\mathrm{mT}$ and magnetic frequency was $f_z = 10\,\mathrm{Hz}$.

Video S6 (.mp4 format)**.** The interfacial instability of swarms consisting of microparticles with different diameters and susceptibilities. The diameters of microparticles used in the experiment were 2, 4, 6, and 8 μm, respectively.

Video S7 (.mp4 format)**.** The morphology manipulation of the unstable interfaces of the microswarms.

Video S8 (.mp4 format)**.** The number manipulation of the unstable interfaces of the microswarms.

Video S9 (.mp4 format)**.** The dynamic manipulation of the unstable interfaces of the microswarms under the dynamic magnetic field. The dynamic magnetic field consisting of an in-plane rotating magnetic field and an oscillating magnetic field along the z-axis. The in-plane rotating magnetic field has a frequency of $f_{\mathrm{xy}} = 1\ \mathrm{Hz}$ and an amplitude of $B_{\mathrm{x}} = B_{\mathrm{y}} = 0.40\ \mathrm{mT}$. The oscillating magnetic field has a frequency of $f_{\mathrm{z}} = 10\ \mathrm{Hz}$ and an amplitude of $B_{\mathrm{z}} = 3.71\ \mathrm{mT}$.

Supplementary Notes

Note S1. Details of the forces exerted on the microswarm in the nodal plane.

The interfacial instability occurred at the two-phase interface between the microswarm and solution was induced by the coupled action of the magnetic and acoustic fields. Therefore, we should consider both the contribution of the magnetic and acoustic fields to the interfacial instability. The induced magnetic dipole of the microparticles under the time-dependent magnetic field can be calculated as follows:

$$m_{\mathrm{p}}(t) = V_{\mathrm{p}}\chi_{\mathrm{p}}B(t)/\mu_0 \tag{1}$$

where $V_{\mathrm{p}} = (4\pi R_{\mathrm{p}}^3)/3$ is the volume of the microparticles, R_{p} is the radius of the microparticles, $\chi_{\mathrm{p}} = 3\chi/(3+\chi)$ is the effective magnetic susceptibility of the microparticles,[1] χ is the magnetic susceptibility of the material, *t* is the time, $\mu_0 = 4\pi\times10^{-7}$ is the vacuum magnetic susceptibility constant, and *B* is the strength of the applied magnetic field. The oscillating magnetic field was applied along the z-axis in the experiment, which can be written as follows:

$$\boldsymbol{B}_{\mathrm{z}}(t) = B_{\mathrm{z}} \cdot \sin\left(2\pi f_{\mathrm{z}} t\right) \boldsymbol{e}_{\mathrm{z}} \tag{2}$$

where B_{z} is the maximum magnetic field strength along the z-axis, f_{z} is the frequency of the oscillating magnetic field, and $\boldsymbol{e}_{\mathrm{z}}$ is the unit vector along the z-axis. The magnetic microparticles within the swarm self-assembled into chain-shaped structures under the action of the magnetic field.[2, 3] The microparticle chains tended to align with the direction of the external magnetic field. Furthermore, the acoustic standing wave field tended to confine the microparticle chains within the nodal plane. Due to the applied magnetic field was an oscillating field, the microparticle chains oscillated in response to changes in the strength of the input magnetic field under the coupled action of the magnetic and acoustic fields. In this case, the solid phase at the interface of the swarm periodically moved in and out of the liquid phase with a certain acceleration, resulting in the formation of local convection. Furthermore, the density of the microparticles was much greater than that of water. Therefore, when the dense phase attempted to displace the light phase, the interfacial instability occurred at the interface of the two phases. This interfacial instability was similar to the secondary waves observed at the interface of a miscible ferrofluid under the action of a vertical magnetic field.[4] Despite differences in particle size and field configuration, the magnetic force density acting on the microswarm under the varying magnetic field can be calculated similarly to that on the ferrofluid when only the magnetic term is considered. Therefore, the magnetic force density acting on the microswarms can be estimated as follows:[5]

$$f_{\mathrm{m}} \sim \frac{\chi_{\mathrm{p}}}{\mu_0}\left(\frac{\mathrm{d}B_{\mathrm{z}}}{\mathrm{d}t}\right)^2 t^2 \tag{3}$$

Due to the propagation of the ultrasonic wave along the radial direction, the acoustic pressure gradient formed in the nodal plane. The unstable interfaces were stabilized by the transverse component of the acoustic radiation force. The acoustic force density acting on the microswarms can be estimated as follows:[6]

$$f_{ac} \sim -\frac{4X_{01}}{D}\varphi_d E_{ac} J_0\left(X_{01}\varphi_h\right) J_1\left(X_{01}\varphi_h\right) \tag{4}$$

where $\varphi_d = 3\left(\rho_p - \rho_m\right)/\left(2\rho_p + \rho_m\right)$ is the acoustic dipole coefficient,[7] ρ_p is the density of the microparticles, ρ_m is the density of the solution, $E_{ac} = p_a^2/4\rho_m c_a^2$ is the acoustic energy density,[8] p_a is the pressure amplitude of the acoustic field, c_a is the speed of sound in water, D is the diameter of the experimental cell, $\varphi_h = \left(2r + D_0\right)/D$ is the height contrast coefficient, r is the instantaneous height of each tip of unstable interfaces, D_0 is the initial diameter of the microswarm under the ultrasonic and static magnetic field along the z-axis, J_m $(m = 0, 1)$ is an m-order Bessel function of the first kind, and X_{01} is the first solution to the equation $J_0\left(x\right) = 0$ (Figure S11). The darg force density acting on the microswarms during the oscillatory process can be estimated as follows:[9]

$$f_d \sim -\frac{9C_m\eta_m(N-1)}{2\rho_p R_p}\omega \tag{5}$$

where C_m is the mass concentration of the solution containing the microparticles, N is the number of microparticles in the individual chain, ω is the rotational angular velocity of the microparticle chains, and η_m is the dynamic viscosity of the solution. According to Newton's second law of motion, the equilibrium relationship between the forces acting on the microswarm in the nodal plane can be expressed as follows:

$$\rho_p \frac{d^2 r}{dt^2} = f_m + f_{ac} + f_d \tag{6}$$

Compared to these forces, the two-phase interfacial tension and diffusion effect are very weak, which can be negligible in our experiment. The two-phase interfacial tension can be calculated as follows:[10]

$$\gamma_{\mathrm{ls}} = \frac{k_{\mathrm{B}}T}{4R_{\mathrm{p}}^2}\left(\frac{C_{\mathrm{m}}}{\rho_{\mathrm{p}}}\right) \sim 4.88\times10^{-17}\ \mathrm{N\cdot m^{-1}} \tag{7}$$

where k_{B} is the Boltzmann constant and T is the temperature. The diffusion coefficient can be calculated as follows:[11]

$$D_{\mathrm{p}} = \frac{k_{\mathrm{B}}T}{6\pi\eta_{\mathrm{m}}R_{\mathrm{p}}} \sim 0.05\ \mathrm{\mu m^2\cdot s^{-1}} \tag{8}$$

Note S2. Magnetic property calculations of the microparticles and the numerical simulation model of the magnetic field.

The magnetic property measurement of the microparticles with different diameters was done using a vibrating-sample magnetometer. During the measurement, the spherical microparticels generated an internal demagnetization field that opposed the applied magnetic field, which reduced the measured susceptibilities. According to the obtained hysteresis curve and magnetic strength used in the experment ($B_{\mathrm{z}} = 3.71\ \mathrm{mT}$), we can calculate the magnetic susceptibility of the microparticles with different diameters (Figure S12). Considering the Clausius–Mossoti function, the effective magnetic susceptibility of the magnetic microparticles can be calculated as follows:[1]

$$\chi_{\mathrm{p}} = \frac{3\chi}{3+\chi} \tag{9}$$

We characterized the current in the coils and the magnetic strength generated by the three-axis Helmholtz coils (Figure S2a) using the galvanometer and teslameter, respectively. The current and magnetic strength at different peak-to-peak voltages of the sinusoidal signal fed into the coils are shown in Figure S2b, c. We further simulated the magnetic field generated by a set of three-axis Helmholtz coils. A finite element model was established using the magnetic fields module of a commercial finite element analysis software (COMSOL 5.5). The domain was modeled using a three-dimensional spherical geometry with the diameter of 200 mm. A set

of three-axis Helmholtz coils was in the center of the spherical domain. The geometric parameters of the Helmholtz coil are shown in Figure S13. The governing equation for computing the magnetic field in the spherical domain is:[12]

$$\nabla \times \boldsymbol{B} = \frac{\mu_0 n I}{A} \boldsymbol{e} \tag{10}$$

where n is the turns number of the coils, I is the amplitude of the current in the coils, A is the cross-sectional area of the wires, and $\boldsymbol{e}$ is the unit vector. In our simulation, the turns number of the coils was 200 and the amplitude of the current was 8 A. Other detailed parameters used in the numerical simulation are listed in Table S1 and S2. The numerical simulation results of the magnetic field generated by the Helmholtz coil is shown in Figure S2d. The numerical simulation results of the magnetic field along the y-axis and z-axis are shown in Figure S2e, f, respectively. It can be known from the numerical simulation results that the magnetic field strength along the radial direction is much smaller than that along the z-axis. Furthermore, the region of the uniform magnetic field in the x-y plane is much larger than the projected area of the microswarm.

Note S3. Details of the torques exerted on the microparticle chains.

Under the oscillating magnetic field, the microparticle chains were subjected to an alternating magnetic torque during the alignment process. The magnetic torque acting on the microparticle chain can be calculated as follows:[13]

$$\Gamma_{\mathrm{m}} = \frac{V_{\mathrm{p}} \chi_{\mathrm{p}}^2 B_{\mathrm{z}}^2 (N-1)}{8\mu_0} \sin(2\alpha) \tag{11}$$

where α is the angle between the microparticle chain and z-axis. The drag torque acting on the microparticle chains during the alignment process can be calculated as follows:[14]

$$\Gamma_{\mathrm{d}} = -2 V_{\mathrm{p}} \eta_{\mathrm{m}} \omega \frac{N^3}{\ln(N/2)} \tag{12}$$

Due to the applied acoustic field, the microparticle chains were also subjected to an acoustic torque during the alignment process. Here, to simplify the calculation, we assume that the number of microparticles in the individual chain (N) is even. The resulting deviation is very small and can be negligible.[15] Therefore, the acoustic torque acting on the microparticle chains can be expressed as follows:[16]

$$\Gamma_{\mathrm{a}} = -\sum_{i=1}^{N/2} 6V_{\mathrm{p}}E_{\mathrm{ac}}k_{\mathrm{a}}\varphi_{\mathrm{c}}R_{\mathrm{p}}\left(2i-1\right)\sin\left(\alpha\right)\sin\left[k_{\mathrm{a}}R_{\mathrm{p}}\left(4i-2\right)\cos\left(\alpha\right)\right] \tag{13}$$

where k_{a} is the wavenumber of the applied ultrasonic wave, $i = 1, 2, \cdots, N/2$ is the positive integer, and φ_{c} is the acoustic contrast factor, which can be written as follows:[16]

$$\varphi_{\mathrm{c}} = \frac{5\rho_{\mathrm{p}} - 2\rho_{\mathrm{m}}}{6\rho_{\mathrm{p}} + 3\rho_{\mathrm{m}}} - \frac{1}{3}\frac{\rho_{\mathrm{m}}c_{\mathrm{a}}^{2}}{\rho_{\mathrm{p}}c_{\mathrm{p}}^{2}} \tag{14}$$

where c_{p} is the speed of sound in the microparticle. When the oscillating magnetic field and acoustic field were simultaneously applied, the microparticle chains exhibited rotational motion behavior under the action of magnetic, acoustic and drag torques. Therefore, the counterbalance relationship between the torques exerted on the microparticle chain can be expressed as follows:

$$I_{\mathrm{p}}\frac{\mathrm{d}\omega}{\mathrm{d}t} = \Gamma_{\mathrm{m}} + \Gamma_{\mathrm{d}} + \Gamma_{\mathrm{a}} \tag{15}$$

where I_{p} is the moment of inertia of the chain.

Note S4. The numerical simulation model of the acoustic field.

We calculated the acoustic pressure, acoustic radiation force and acoustic streaming fields in the nodal plane of the acoustic platform through the finite element simulation method. A finite element model was established using the commercial finite element analysis software (COMSOL 5.5) with fully coupled pressure acoustics and creeping flow modules. The three-dimensional simulation domain consisted of four parts: the experimetal cell, polyimide tape,

silicon wafer and ultrasonic transducer (Figure S6a). The detailed material and geometric parameters used in the numerical simulation are listed in Table S1 and S2. The governing equation for simulating the acoustic pressure field in the domain can be expressed as follows:[17]

$$\nabla^2 p + \left(\frac{\omega_\mathrm{a}}{c}\right)^2 p = 0 \tag{16}$$

where p is the acoustic pressure, ω_a is the angular frequency, and c is the wave speed in the domain. We used the quadratic Lagrangian shape functions to simulate the acoustic pressure fields. The upper surfaces of the experimetal cell and polyimide tape were set as the acoustic hard boundaries, and other boundaries of the domain were set as the acoustic soft boundaries.[18] Furthermore, the acoustic velocity in the domain can be calculated as follows:[19]

$$v = -\frac{\nabla p}{j\omega_\mathrm{a}\rho} \tag{17}$$

where ρ is the density and j is the imaginary unit. According to the simulated acoustic pressure and velocity, the acoustic radiation force acting on the magnetic microparticles can be calculated as follows:[20]

$$F_\mathrm{rad} = -\nabla \left\{ \frac{V_\mathrm{p}}{2} \left[\left(1 - \frac{\rho_\mathrm{m} c_\mathrm{a}^2}{\rho_\mathrm{p} c_\mathrm{p}^2} \right) \frac{\langle p \rangle^2}{\rho_\mathrm{m} c_\mathrm{a}^2} - \varphi_\mathrm{d} \rho_\mathrm{m} \langle v \rangle^2 \right] \right\} \tag{18}$$

where $\langle X \rangle = (1/\tau) \cdot \int_0^\tau X(t)\,\mathrm{d}\tau$ denotes time averaging. The governing equations for simulating the acoustic streaming field in the fluid domain can be expressed as follows:

$$\nabla \cdot \boldsymbol{v}_\mathrm{m} = 0 \tag{19}$$

$$\rho_\mathrm{m} (\boldsymbol{v}_\mathrm{m} \cdot \nabla) \boldsymbol{v}_\mathrm{m} = \nabla \cdot \left\{ -p_\mathrm{m} \boldsymbol{E} + \eta_\mathrm{m} \left[\nabla \boldsymbol{v}_\mathrm{m} + (\nabla \boldsymbol{v}_\mathrm{m})^\mathrm{T} \right] \right\} + \boldsymbol{F}_\mathrm{b} \tag{20}$$

$$\boldsymbol{F}_\mathrm{b} = -\rho_\mathrm{m} \left\langle (\boldsymbol{v} \cdot \nabla) \boldsymbol{v} + \boldsymbol{v} (\nabla \cdot \boldsymbol{v}) \right\rangle \tag{21}$$

where $\boldsymbol{v}_\mathrm{m}$ is the acoustic streaming velocity, p_m is the pressure in the fluid domain, $\boldsymbol{E}$ is the identity matrix, $\boldsymbol{F}_\mathrm{b}$ is the body force induced by the acoustic field.[21, 22] The second order

discretization for the velocity field and first order discretization for the pressure field were used for the fluid flow. The boundaries of the fluid domain were set to the no-slip boundaries.

In a typical process, to generate the ultrasonic standing wave in the experimental cell, polyimide tapes with a thickness of 250 μm and the ultrasonic wave with a frequency of 3 MHz were used in the experiment. Therefore, we simulated the acoustic pressure, acoustic radiation force and acoustic streaming fields in the nodal plane of the experimental cell at the acoustic frequency of 3 MHz. The numerical simulation results are shown in Figure S6b-d, respectively. It can be seen from the simulation results that the microparticles move towards the acoustic pressure antinodes in the nodal plane due to the in-plane acoustic radiation and acoustic streaming that point to the antinodes.[23, 24] We have further investigated the influence of the ultrasonic field on the morphology and number of the unstable interfaces using the finite element simulation method. When simulating the effect of the acoustic field on the morphology of the unstable interfaces, the shapes of the polyimide tape were changed into the square, triangle, crescent, and bow tie (Figure S7 and S8). Otherwise, the conditions remained unchanged. When simulating the effect of the acoustic field on the number of the unstable interfaces, the conditions remained unchanged, except that the height of the ring-shaped polyimide tape was changed into 375 μm and 150 μm, corresponding to the ultrasonic frequencies of 2 MHz and 5 MHz, respectively (Figure S9). Despite the distribution of the acoustic pressure field was changed by tuning the shape and height of the polyimide tapes, the microparticles still moved towards the acoustic pressure antinodes, which was independent of the acoustic pressure field distribution.

Note S5. Details of the thermal field distribution simulation of the microswarms.

We have simulated the thermal field distribution of circular- and gear-shaped microswarms. In the absence of phase transformations, the thermal field distribution around the microswarms irradiated by the near-infrared light can be calculated by the heat transfer equation as follows:[25]

$$\rho(x)c_{\mathrm{h}}(x)\partial_t T(x,t)=\nabla k(x)\nabla T(x,t)+Q(x,t) \tag{22}$$

where x is the coordinate, $\rho(x)$ is the mass density, $c_{\mathrm{h}}(x)$ is the specific heat, $T(x,t)$ is the local temperature, $k(x)$ is the thermal conductivity of the material, and $Q(x,t)$ is the local heat intensity resulting from the laser irradiation. We can further calculate the local temperature change around the microswarms in the steady state:[26]

$$\Delta T=\frac{V_{\mathrm{n}}\omega_{\mathrm{e}}}{32\pi^2 k_{\mathrm{m}}d}E_0^2\left|\frac{3\varepsilon_{\mathrm{m}}}{2\varepsilon_{\mathrm{m}}+\varepsilon_{\mathrm{n}}}\right|^2\mathrm{Im}(\varepsilon_{\mathrm{n}}) \tag{23}$$

where $V_{\mathrm{n}}=4\pi R_{\mathrm{n}}^3/3$ is the volume of the Au nanoparticles, R_{n} is the radius of the Au nanoparticles, ω_{e} is the frequency of the resulting electric field, k_{m} is the thermal conductivity of the medium, d is the distance from the center of the microparticles, ε_{m} is the dielectric constants of the medium, ε_{n} is the dielectric constants of Au nanoparticles, $E_0=\sqrt{2I_1/(n_{\mathrm{m}}\varepsilon_{\mathrm{m}}c_1)}$ is the amplitude of the incident light, I_1 is the light intensity, n_{m} is the refractive index of the medium, c_1 is the speed of light in vacuum, and Im( ) denotes the imaginary part.

Supplementary Figures

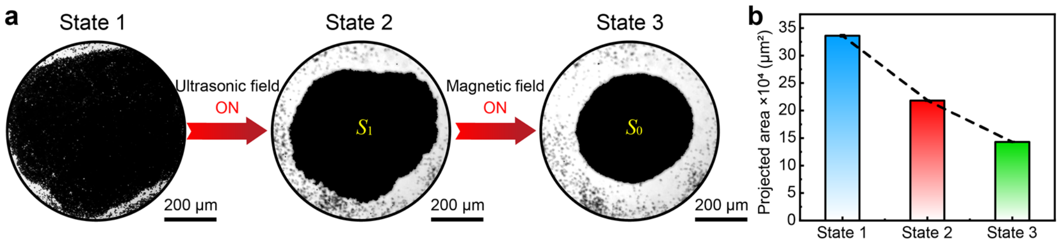

Figure S1. The changes in the projected area of the microswarm during the formation process of the circular interface. a) The microparticles have three states during the formation of the circular interface. State 1: no external fields was applied, microparticles were uniformaly dispersed in the solution. State 2: an ultrasonic field was applied, microparticles moved toward the pressure node of the standing wave and aggregated into the swarm with a projected area of S_1 . State 3: the ultrasonic and static or high frequency (30 Hz) magnetic fields were simultaneously applied, the microparticles were further agglomerated with a projected area of S_0 . Scale bar: 200 μm. b) The changes in the covered projected area of microparticles under different states. The sinusoidal signal with a frequency of $f_a = 3\,\text{MHz}$ and a peak-to-peak voltage of $V_{ac} = 10\,\text{V}$ was fed into the ultrasonic transducer to generate the ultrasonic field. The amplitude of the oscillating magnetic field was $B_z = 3.71\,\text{mT}$.

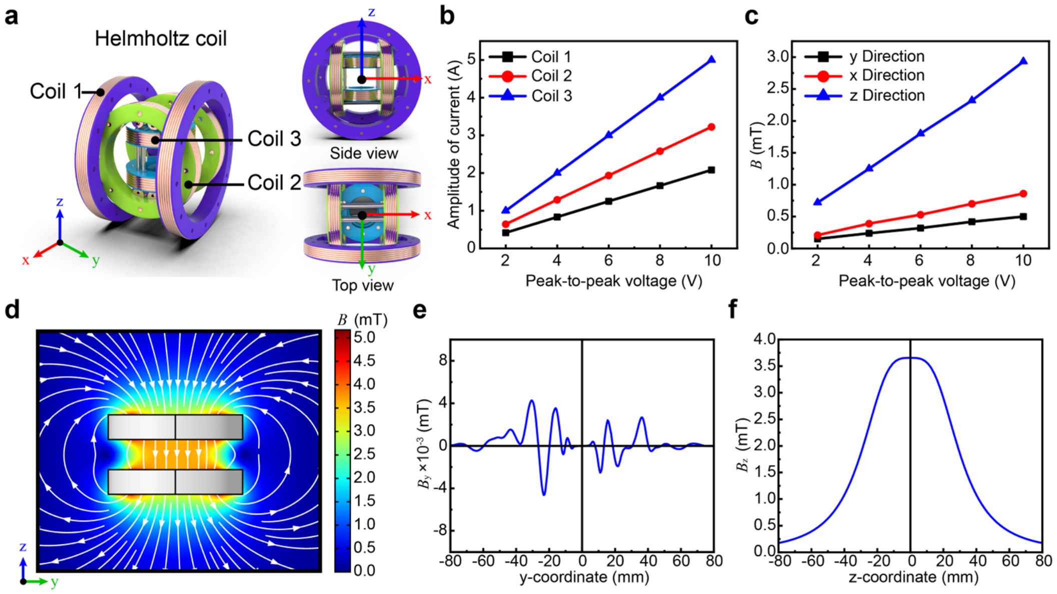

Figure S2. Schematic illustration of three-axis Helmholtz coils and characterization of the magnetic field. a) Schematic illustration of three-axis Helmoholtz coils used in the experiment. b) The amplitude of the current in coils versus the peak-to-peak voltage of the signal fed into coils. c) The amplitude of the magnetic field B along three different directions versus the peak-to-peak voltage of the signal fed into coils. d) The numerical simulation results of the magnetic field strength generated by a set of three-axis Helmoholtz coils. The white lines represent the magnetic field lines. e) The amplitude of the magnetic field along the y-axis B_y. f) The amplitude of the magnetic field along the z-axis B_z.

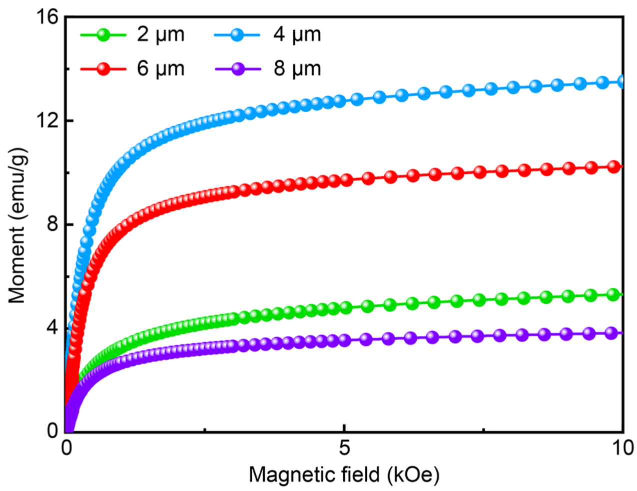

Figure S3. The initial magnetization curve of microparticles with different diameters.

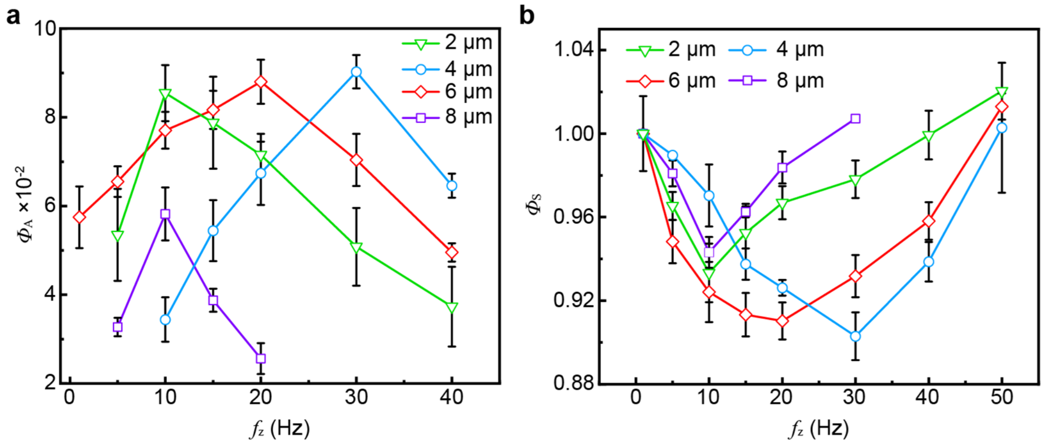

Figure S4. The influence of the magnetic frequency on the unstable interface and the morphology of the swarm consisting of different microparticles. a) The normalized characteristic height ϕ_A of unstable interfaces versus the magnetic frequency f_z. The magnetic field strength was $B_z = 3.71\,\mathrm{mT}$ and microparticle diameters used in the experiment were 2, 4, 6 and 8 μm. b) The normalized projected area ϕ_S of swarms versus the magnetic frequency f_z. The magnetic field strength was $B_z = 3.71\,\mathrm{mT}$ and microparticle diameters used in the experiment were 2, 4, 6 and 8 μm.

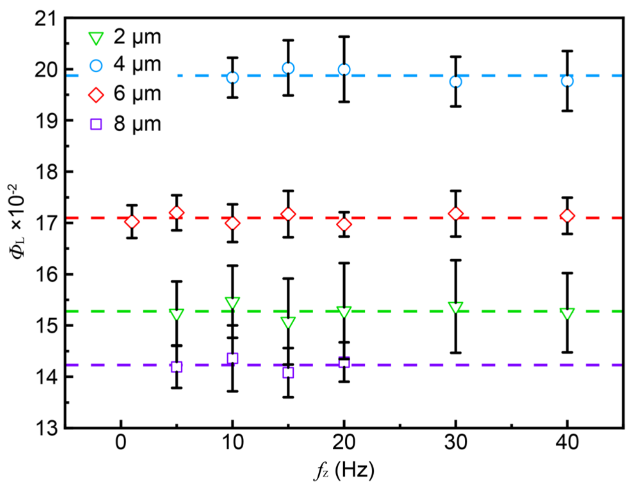

Figure S5. The normalized characteristic wavelength of unstable interfaces versus the magnetic frequency, where microparticle diameters are 2, 4, 6 and 8 μm.

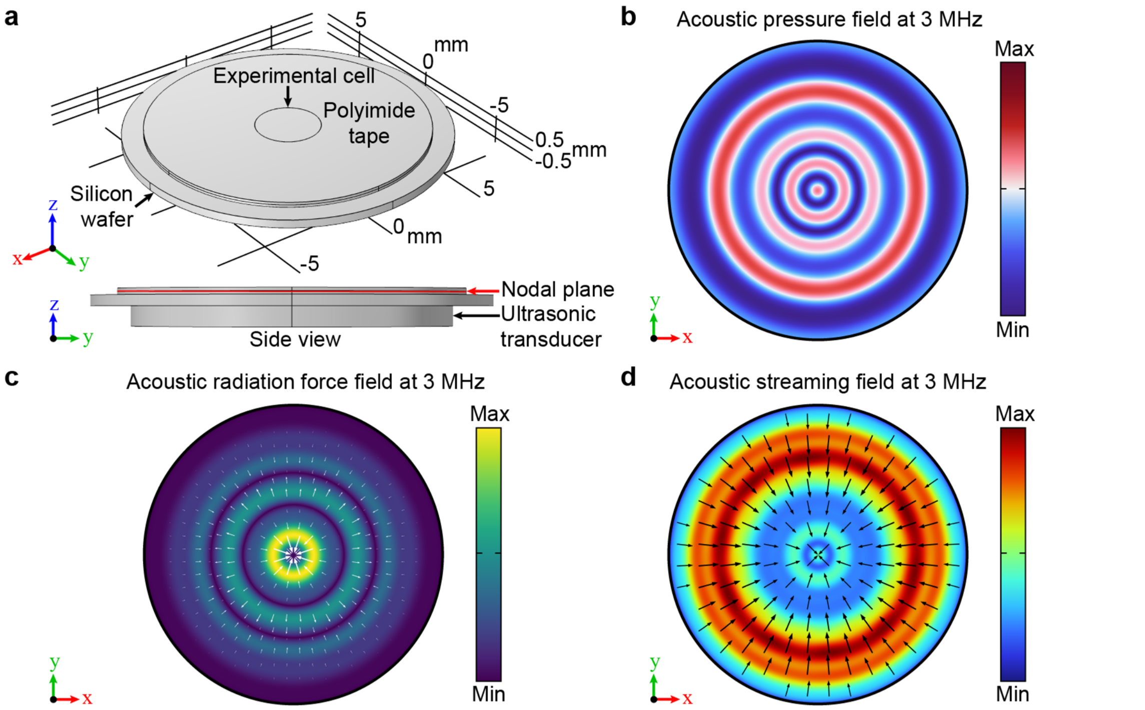

Figure S6. Illustration of the acoustic simulation model and the simulated acoustic pressure, acoustic radiation force and acoustic streaming fields at an acoustic frequency of 3 MHz. a) The three-dimensional simulation domain used for the finite element simulation of the acoustic field. b) The numerical simulation results of the acoustic pressure field at 3 MHz in the nodal plane. c) The numerical simulation results of the acoustic radiation force field at 3 MHz in the nodal plane. The white arrows represent directions of acoustic radiation forces. d) The numerical simulation results of the acoustic streaming field at 3 MHz in the nodal plane. The black arrows represent directions of the acoustic streaming.

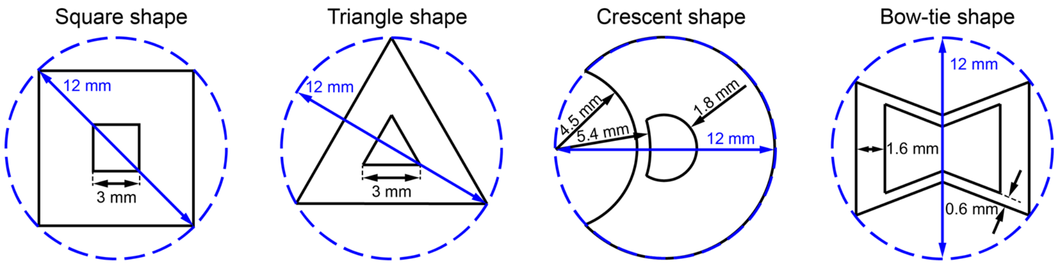

Figure S7. The geometric parameters of different polyimide tapes used in the experiment.

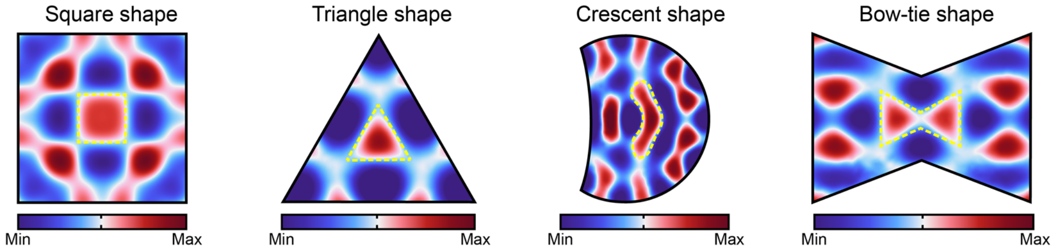

Figure S8. Numerical simulation results of the acoustic pressure field in the experimental cell nodal plane for different shapes of the polyimide tapes.

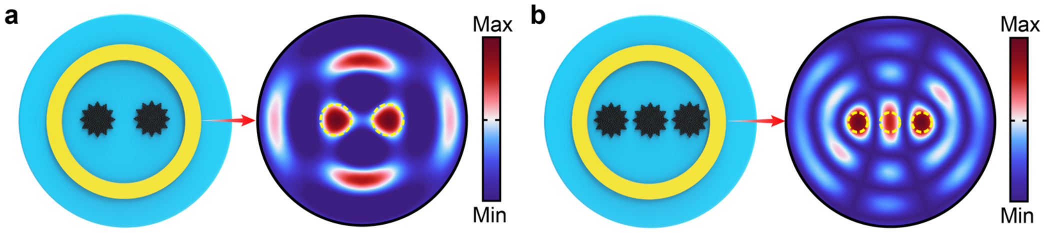

Figure S9. Numerical simulation results of the number manipulation of the unstable interfaces. a) Simulation results of the acoustic pressure field show that two acoustic pressure nodes are formed in the center of the nodal plane. The height of the polyimide tape was $h = 375\ \mu\text{m}$, and the ultrasonic frequency was $f_a = 2\ \text{MHz}$. b) Simulation results of the acoustic pressure field show that three acoustic pressure nodes are formed in the center of the nodal plane. The height of the polyimide tape was $h = 150\ \mu\text{m}$, and the ultrasonic frequency was $f_a = 5\ \text{MHz}$.

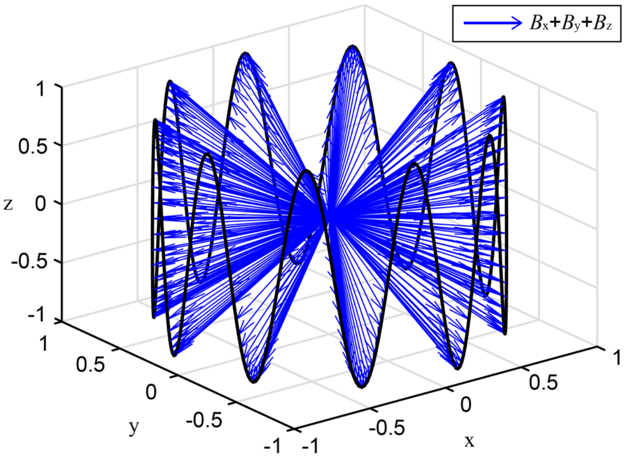

Figure S10. Visualization of the dynamic magnetic field. The magnetic strength was normalized.

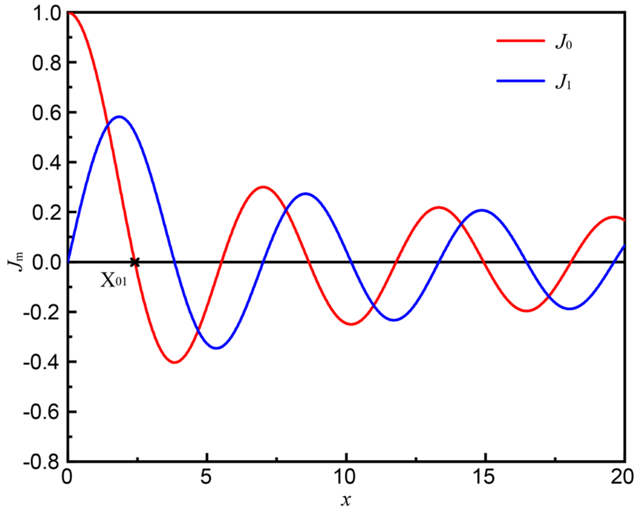

Figure S11. The zero-order (J_0) and first-order (J_1) Bessel function of the first kind. X_{01} represents the first solution of the zero-order Bessel function of the first kind.

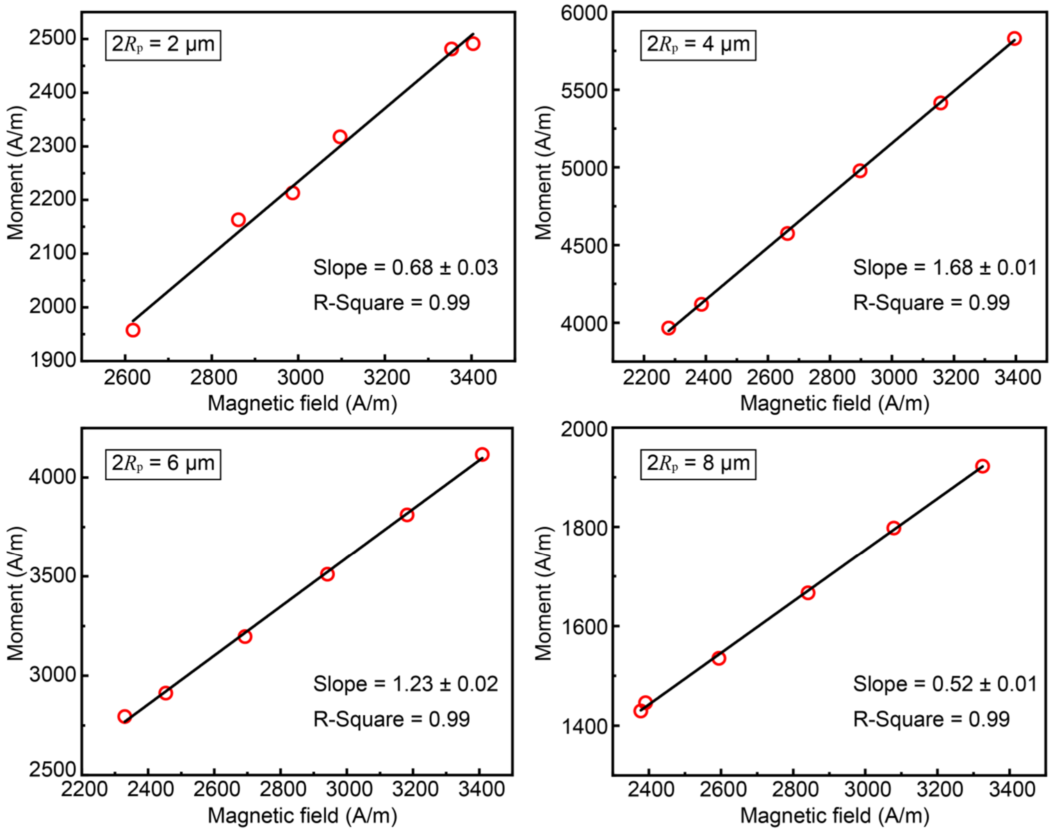

Figure S12. The linear fit of the magnetic property measurement data for microparticles with different diameters reveals the magnetic susceptibility from the slope.

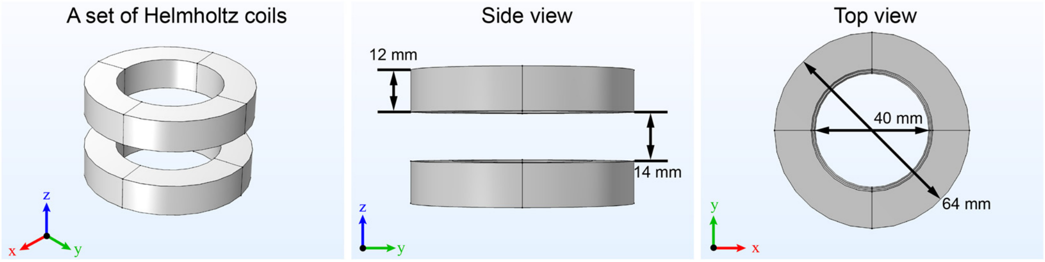

Figure S13. The geometric parameters of the Helmholtz coil used in the magnetic simulation.

Supplementary Tables

Table S1. Material and field parameters used for the numerical simulations.

**Water**	
Density	1000 kg/m^3
Sound speed	1500 m/s
Dynamic viscosity	1.01×10^{-3} Pa·s
**Silicon wafer**	
Density	2203 kg/m^3
Sound speed	8490 m/s
**Polyimide tape**	
Density	1300 kg/m^3
Sound speed	2200 m/s
**Magnetic microparticles**	
Diameter	2 μm
Density	5180 kg/m^3
Compressibility	1.72×10^{-10} 1/Pa
Sound speed	6760 m/s
**Au nanoparticles**	
Diameter	20 nm
Dielectric constant	-5.614 + 2.256i
**Ultrasonic field**	
Frequency	3 MHz

Magnetic field	
Magnetic susceptibility of vacuum	$4\pi \times 10^{-7}$ N/A^2
**Light field**	
Wavelength	808 nm
Intensity	100 W/cm^2
Speed	3×10^8 m/s
**Thermal field**	
Conductivity	0.1 W/(m·K)
Refractive index	1.456
**Helmholtz coil**	
Number of turns	200
Amplitude of current	8 A
Conductivity of wire	5×10^7 S/m
Cross-sectional area of wire	0.28 mm^2

Table S2. Geometric parameters used for the numerical simulations.

Helmholtz coil	
Cross-sectional area	12 mm × 12 mm
Inner diameter	40 mm
Outer diameter	64 mm
Distance between two coils	14 mm
**Acoustic platform**	
Diameter of experimental cell	3 mm
Height of experimental cell	0.25 mm
Diameter of polyimide tape	12 mm
Height of polyimide tape	0.25 mm
Diameter of silicon wafer	15 mm
Height of silicon wafer	0.4 mm
Diameter of ultrasonic transducer	12 mm
Height of ultrasonic transducer	0.75 mm

Supplementary References

[1] Y. Gao, M. Hulsen, T. Kang, J. Den Toonder, *Phys. Rev. E* **2012**, *86*, 041503.

[2] J. F. Yu, B. Wang, X. Z. Du, Q. Q. Wang, L. Zhang, *Nat. Commun.* **2018**, *9*, 3260.

[3] Q. Q. Wang, J. F. Yu, K. Yu, L. D. Yang, D. D. Jin, L. Zhang, *Appl. Mater. Today* **2020**, *18*, 100489.

[4] C. Y. Wen, C. Y. Chen, D. C. Kuan, *Phys. Fluids* **2007**, *19*, 084101.

[5] C. Y. Wen, J. Z. Lin, M. Y. Chen, L. Q. Chen, T. K. Liang, *J. Magn. Magn. Mater.* **2011**, *323*, 1258.

[6] G. Whitworth, W. Coakley, *J. Acoust. Soc. Am.* **1992**, *91*, 79.

[7] S. M. Woodside, B. D. Bowen, J. M. Piret, *AICHE J.* **1997**, *43*, 1727.

[8] H. Bruus, *Lab Chip* **2012**, *12*, 1014.

[9] Z.-C. Zhang, Y.-P. Li, L.-J. Zhang, D.-X. Chen, *J. Fluid Struct.* **2023**, *118*, 103861.

[10] P. van der Schoot, *J. Phys. Chem. B* **1999**, *103*, 8804.

[11] J. R. Howse, R. A. Jones, A. J. Ryan, T. Gough, R. Vafabakhsh, R. Golestanian, *Phys. Rev. Lett.* **2007**, *99*, 048102.

[12] C. Jiang, P. Gao, X. Yang, D. Ji, J. Sun, Z. Yang, *Appl. Sci.* **2022**, *12*, 9832.

[13] I. Petousis, E. Homburg, R. Derks, A. Dietzel, *Lab Chip* **2007**, *7*, 1746.

[14] C. Wilhelm, J. Browaeys, A. Ponton, J.-C. Bacri, *Phys. Rev. E* **2003**, *67*, 011504.

[15] T. Franke, L. Schmid, D. A. Weitz, A. Wixforth, *Lab Chip* **2009**, *9*, 2831.

[16] W. Wang, W. Duan, S. Ahmed, T. E. Mallouk, A. Sen, *Nano Today* **2013**, *8*, 531.

[17] L. E. Kinsler, A. R. Frey, A. B. Coppens, J. V. Sanders, *Fundamentals of acoustics*, Wiley, **2000**.

[18] Q. Tang, J. Hu, *Microfluid. Nanofluid.* **2015**, *19*, 1395.

[19] D. T. Blackstock, *Fundamentals of physical acoustics*, Wiley, **2001**.

[20] L. P. Gor'kov, *Sov. Phys.-Doklady* **1962**, *6*, 773.

[21] W. L. Nyborg, *J. Acoust. Soc. Am.* **1953**, *25*, 68.

[22] J. Lighthill, *J. Sound Vib.* **1978**, *61*, 391.

[23] J. Lei, *Microfluid. Nanofluid.* **2017**, *21*, 50.

[24] G. Vuillermet, P.-Y. Gires, F. Casset, C. Poulain, *Phys. Rev. Lett.* **2016**, *116*, 184501.

[25] A. O. Govorov, W. Zhang, T. Skeini, H. Richardson, J. Lee, N. A. Kotov, *Nanoscale Res. Lett.* **2006**, *1*, 84.

[26] A. O. Govorov, H. H. Richardson, *Nano today* **2007**, *2*, 30.
